# Chromosomal Instability by Inefficient Mps1 Auto-Activation Due to a Weakened Mitotic Checkpoint and Lagging Chromosomes

**DOI:** 10.1371/journal.pone.0002415

**Published:** 2008-06-11

**Authors:** Nannette Jelluma, Arjan B. Brenkman, Ian McLeod, John R. Yates, Don W. Cleveland, René H. Medema, Geert J. P. L. Kops

**Affiliations:** 1 Department of Physiological Chemistry and Cancer Genomics Centre, UMC Utrecht, Utrecht, The Netherlands; 2 Department of Medical Oncology, Laboratory of Experimental Oncology, UMC Utrecht, Utrecht, The Netherlands; 3 Department of Cell Biology, The Scripps Research Institute, La Jolla, California, United States of America; 4 Ludwig Institute for Cancer Research and Department of Cellular and Molecular Medicine, University of California San Diego, La Jolla, California, United States of America; University of Edinburgh, United Kingdom

## Abstract

**Background:**

Chromosomal instability (CIN), a feature widely shared by cells from solid tumors, is caused by occasional chromosome missegregations during cell division. Two of the causes of CIN are weakened mitotic checkpoint signaling and persistent merotelic attachments that result in lagging chromosomes during anaphase.

**Principal Findings:**

Here we identify an autophosphorylation event on Mps1 that is required to prevent these two causes of CIN. Mps1 is phosphorylated in mitotic cells on at least 7 residues, 4 of which by autophosphorylation. One of these, T676, resides in the activation loop of the kinase domain and a mutant that cannot be phosphorylated on T676 is less active than wild-type Mps1 but is not kinase-dead. Strikingly, cells in which endogenous Mps1 was replaced with this mutant are viable but missegregate chromosomes frequently. Anaphase is initiated in the presence of misaligned and lagging chromosomes, indicative of a weakened checkpoint and persistent merotelic attachments, respectively.

**Conclusions/Significance:**

We propose that full activity of Mps1 is essential for maintaining chromosomal stability by allowing resolution of merotelic attachments and to ensure that single kinetochores achieve the strength of checkpoint signaling sufficient to prevent premature anaphase onset and chromosomal instability. To our knowledge, phosphorylation of T676 on Mps1 is the first post-translational modification in human cells of which the absence causes checkpoint weakening and CIN without affecting cell viability.

## Introduction

Aneuploidy is a trait widely observed in tumor cells from all types [1]. Aneuploidy in these cells is the result of occasional chromosome missegregations, a phenotype referred to as chromosomal instability or CIN. Various defects in the processes that control chromosome segregation and subsequent cell division can cause CIN [2] and these include but are not limited to tetraploidization, defective mitotic checkpoint function and unresolved merotelic chromosome attachments [3]–[5].

The mitotic checkpoint prevents chromosomal instability by delaying anaphase onset until all chromosomes are correctly attached to the mitotic spindle. To ensure faithful chromosome segregation, the mitotic checkpoint has to be sufficiently strong to delay anaphase when as little as one chromosome is not correctly attached to the spindle. Weakening of the mitotic checkpoint promotes aneuploidy and it has been proposed that this can contribute to tumorigenesis [2], [5]. Recent examination of a number of studies that have investigated mitotic checkpoint responses in a wide variety of tumor cells indicated that about two-thirds of those tumors have a reduced capacity to maintain a mitotic arrest when challenged with spindle poisons [5]. Despite many investigations, molecular explanations for such checkpoint weakening have yet to be found. Genes encoding checkpoint components are very infrequently mutated (reviewed in [2], [5]) and, although reported in some instances, altered expression of checkpoint proteins or mRNA does not appear to correlate well with the status of CIN (e.g. [6]–[8]). It is therefore likely that checkpoint activity is affected by other means, one of which may be a change in the level of functionally relevant post-translational modifications or by misregulated enzymatic activities that are crucial for checkpoint signaling. Finding the relevant modifications that contribute to checkpoint signal strength and investigating their status in chromosomally instable tumor cells might give important information on how checkpoint signaling may have become compromised in those tumors.

Faithful chromosome segregations requires all chromosomes to be bi-oriented, with each sister kinetochore attached to one pole. Most erroneous attachments are sensed, directly or indirectly, by the mitotic checkpoint as they involve lack-of-attachment or lack-of-tension, and are corrected by the chromosomal passenger complex of which Aurora B is the effector kinase [9]. Merotelic attachments, however, escape detection by the mitotic checkpoint. A chromosome is attached in a merotelic fashion when it is bi-oriented while one of its kinetochores has attachments to both poles. This chromosome is therefore attached as well as under tension. Merotely has been suggested as a frequent cause of CIN in tumor cells [3], [10]. Interestingly, slight inhibition of Aurora B caused frequent persistent merotelic attachments that resulted in lagging chromosomes in anaphase and aneuploidy of the resulting daughter cells [11], [12]. Reduced Aurora B activity may therefore be an important cause of chromosome missegregations and CIN in tumor cells.

Monopolar spindle 1 (Mps1) is a kinase that is essential for the mitotic checkpoint as well as for efficient Aurora B activation to allow attachment-error correction [13]–[16]. Despite its central role in ensuring faithful chromosome segregation, little is known about how Mps1 is activated in mitosis or how Mps1 activity correlates with chromosomal stability.

## Results

**Figure 1:**
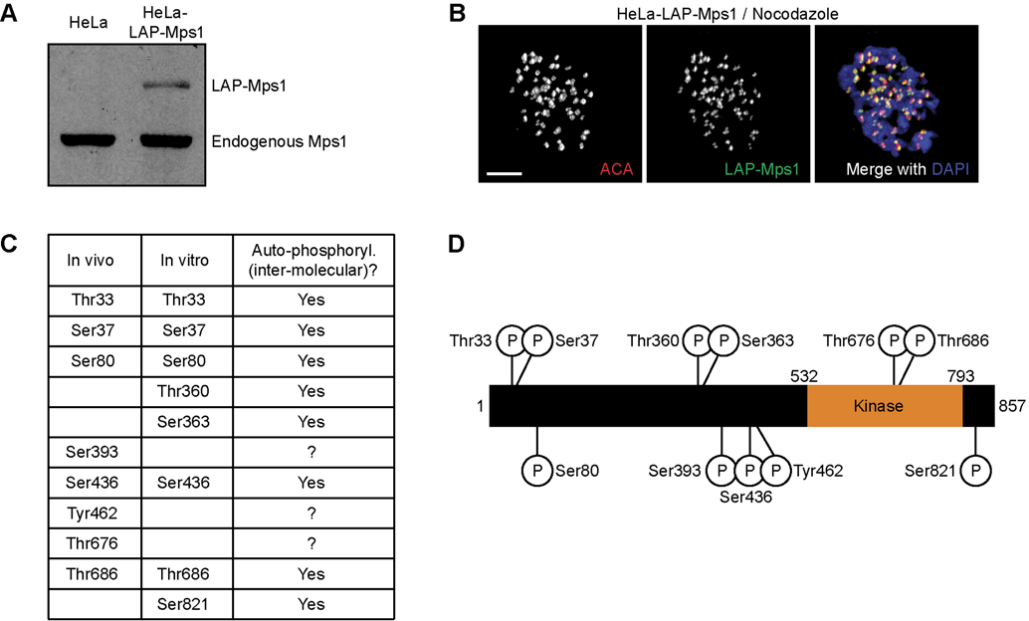
Mps1 is phoshorylated during mitosis on at least 7 residues. (A, B) A monoclononal Hela cell line stably expressing LAP-tagged Mps1 (HeLa-LAP-Mps1) was analyzed for expression level of LAP-Mps1 relative to endogenous Mps1 as shown by immunoblot (anti-Mps1) (A) and localization of LAP-Mps1 (anti-GFP) together with centromeres (ACA) and DNA (DAPI) (B). Scale bar is 5 µm. (C) List of phosphorylation sites identified by mass spectrometry on LAP-Mps1 isolated from mitotic HeLa-LAP-Mps1 cells (left column, in vivo) or on recombinant Mps1 isolated from insect cells (middle column, in vitro). Phosphorylation sites on recombinant Mps1 were designated ‘auto-phosphorylations’ when sites were not found on similarly expressed and isolated recombinant kinase-dead Mps1. (D) Schematic overview of phosphorylated residues on Mps1.

Mps1 is hyperphosphorylated during mitosis, coinciding with a peak in its enzymatic activity [16]. To get insight into the mechanisms of activation of Mps1, mitotic Mps1 was analyzed for the presence of phosphorylated residues. To this end, a HeLa cell line stably expressing low levels of LAP-tagged (Localization and Affinity Purification [17]) Mps1 was used (Fig. 1A, 1B). LAP-Mps1 was isolated from mitotic cells by tandem affinity purification and analyzed for phospho-peptides using mass spectrometry. A total of 8 phosphorylated residues were found (Fig. 1C). In agreement with a recent study [18], one of the identified phospho-residues, T676, is located in the activation (T)-loop of the kinase domain (Fig. 1D). Another potential T-loop phosphorylation on T686 was identified, but confidence of assignment to this site was quite low (see more on this below). Similar identification of phosphorylation sites on in vitro auto-phosphorylated recombinant GST-Mps1 purified from insect cells (see Fig. S1A) revealed that three additional residues were in vitro phosphorylated, and that five of the residues found phosphorylated in vivo, including T686, were the result of auto-phosphorylation (Fig.1C). In agreement with this, T676 and T686 were recently found phosphorylated on bacterially expressed recombinant Mps1 [19].

**Figure 2:**
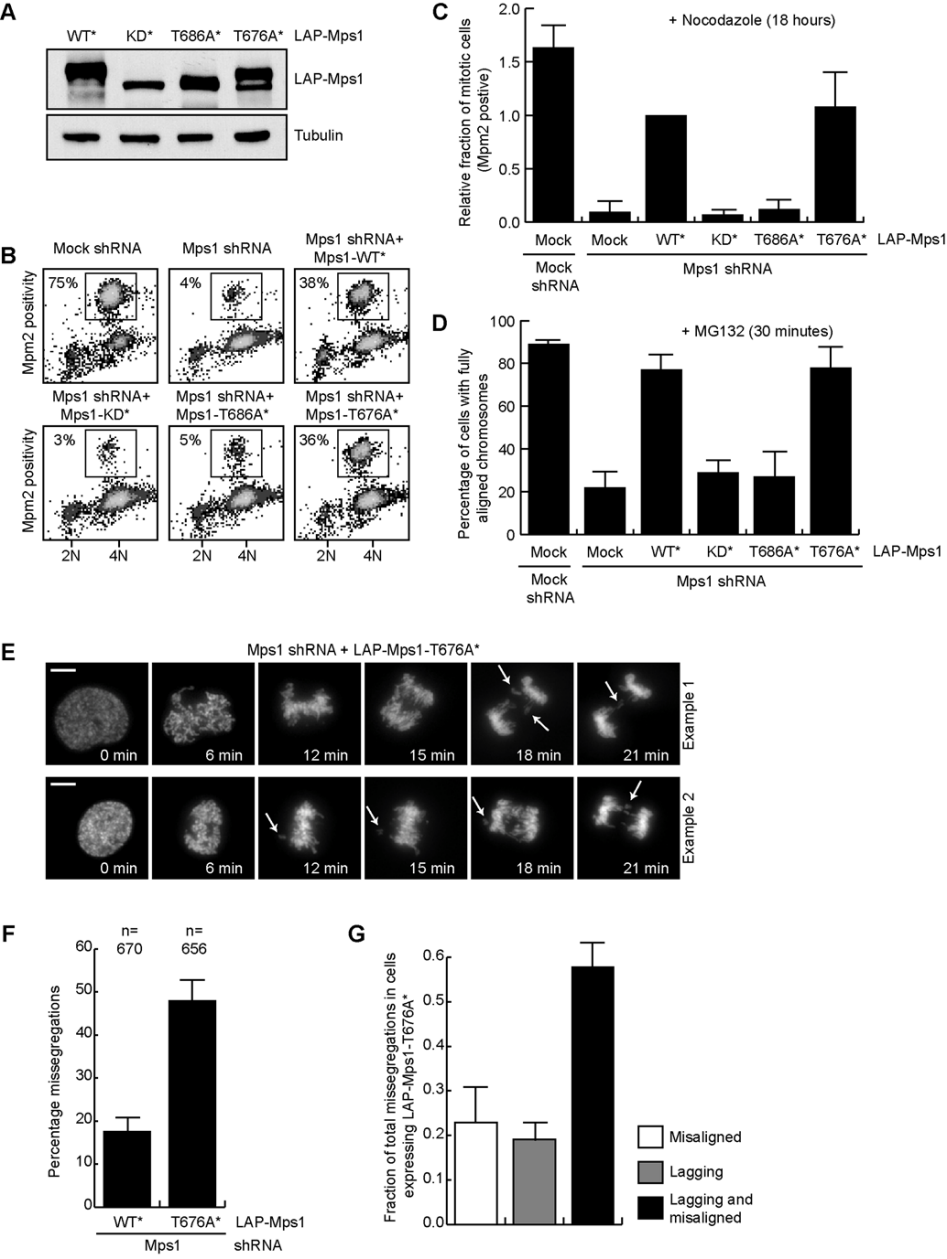
T676 phosphorylation is required for correct chromosome segregation and full checkpoint signaling. (A) Expression levels, as shown by immunoblot (anti-Mps1), in asynchronously growing cells of LAP-Mps1 mutants (asterisk (*) indicates mutations that confer resistance to Mps1 shRNA) transiently transfected into U2OS cells. (B) Flow cytometric analysis of the fraction of mitotic cells transfected with Mock or Mps1 shRNA together with the indicated plasmids (asterisk (*) indicates mutations that confer resistance to Mps1 shRNA), and treated with nocodazole for 18 hrs. (C) Quantification of (B), average of three experiments (+/−SD). (D) U2OS cells transfected as in 2B were treated for 30 minutes with MG132. Fixed cells were stained with DAPI and the percentage of cells with all chromosomes aligned to the metaphase plate was determined (n = 30 cells). Graph represents the average of three experiments (+/−SD). (E) Mitotic progression of U2OS cells transfected as in 2B along with H2B-EYFP Mps1 was followed by live cell imaging. Stills from two representative cells showing anaphase with misaligned/lagging chromosomes (arrows) are shown (see Movie S1 and Movie S2). Scale bar is 5 µm. (F) Quantification of (E), average of three experiments (+/−SD), n = total amount of cells. (G) Graph showing the fraction of total missegregations as displayed in 2F with misaligned chromosomes, lagging chromosomes, or both in cells expressing Mps1-T676A.

Many protein kinases are activated by phosphorylation on T-loop residues [20]. Since both T676 and T686 were reported recently to contribute to kinase activity [18], [19], we created Threonine-to-Alanine mutants of these two sites (T676A and T686A) to study their contribution to mitotic checkpoint signaling, attachment-error-correction and chromosome segregation. To this end, endogenous Mps1 was depleted from U2OS cells using shRNA and replaced with either LAP-tagged wild-type (WT) Mps1 or the T676A or T686A mutants (for details on this assay, see [14]). A kinase-dead (KD) mutant (D664A) was used as negative control. Expression levels of the different mutants are shown in Figure 2A. As expected, depletion of Mps1 prevented cells from accumulating in mitosis upon nocodazole treatment, indicative of a dysfunctional mitotic checkpoint, and caused defects in chromosome alignment. The T686A mutant, like the D664A mutant, was unable to restore either checkpoint signaling (Fig. 2B, 2C) or chromosome alignment (Fig. 2D). The position of this threonine in the kinase domain is very well conserved amongst kinases. It is thought to play a structural role in organizing the catalytic region [21], but in certain kinases, phosphorylation of this site was shown to be necessary for full kinase activity [22]. To discriminate between whether T686A was non-functional due to compromised structural integrity of the kinase domain or due to lack of phosphorylation, a mutant bearing a phospho-mimetic mutation on this site (Aspartic Acid or Glutamic Acid) was tested. Neither Mps1-T686D nor -T686E could restore spindle checkpoint function (Fig. S1B). Even though it is formally possible that the D/E substitutions do not mimic phosphorylation on this site, these data are more consistent with a structural role for T686. In support of this, we have been unable to detect T686 phosphorylation in tissue culture cells using a phospho-specific antibody, even though this antibody was able to recognize in vitro autophosphorylated Mps1 (data not shown).

In contrast to T686A, the T676A mutant restored proper chromosome alignment (Fig. 2D) to the same extent as wild-type. This indicated that attachment-error-correction by Aurora B was not affected, in contrast to cells devoid of Mps1 activity ([14], see discussion for more details). The T676A mutant also rescued mitotic checkpoint signaling in cells treated with nocodazole (Fig. 2B, 2C) to the same extent as wild-type Mps1. Nocodazole treatment presumably causes maximal mitotic checkpoint signaling due to the fact that all kinetochores are unattached. Therefore, defects that do not cause complete inactivation of the checkpoint may not be identified in assays in which nocodazole is used, such as the experiment in Figure 2B. Since the mitotic checkpoint evolved to prevent the missegregation of one or a few chromosomes and therefore requires maximal activity per kinetochore, the contribution of phosphorylation on T676 was studied in more detail by following unperturbed mitotic progression of Mps1-depleted U2OS cells expressing LAP-Mps1-T676A using fluorescence timelapse microscopy. DNA was visualized by co-transfecting H2B-EYFP. 48% (312 out of 656) of cells expressing T676A exited mitosis with one or more lagging and/or misaligned chromosomes, compared to 18% of controls cells (Fig. 2E, 2F, Movie S1, S2). Of cells that missegregated chromosomes, 58% entered anaphase with both misaligned and lagging chromosomes, while 23% and 19% displayed either misaligned or lagging chromosomes, respectively (Fig. 2G). This strongly suggested that cells expressing Mps1-T676A have a diminished capacity to i) delay anaphase onset when few chromosomes are unaligned and ii) resolve misattachments that are not sensed by the mitotic checkpoint. This, in turn, promotes chromosomal instability.

**Figure 3:**
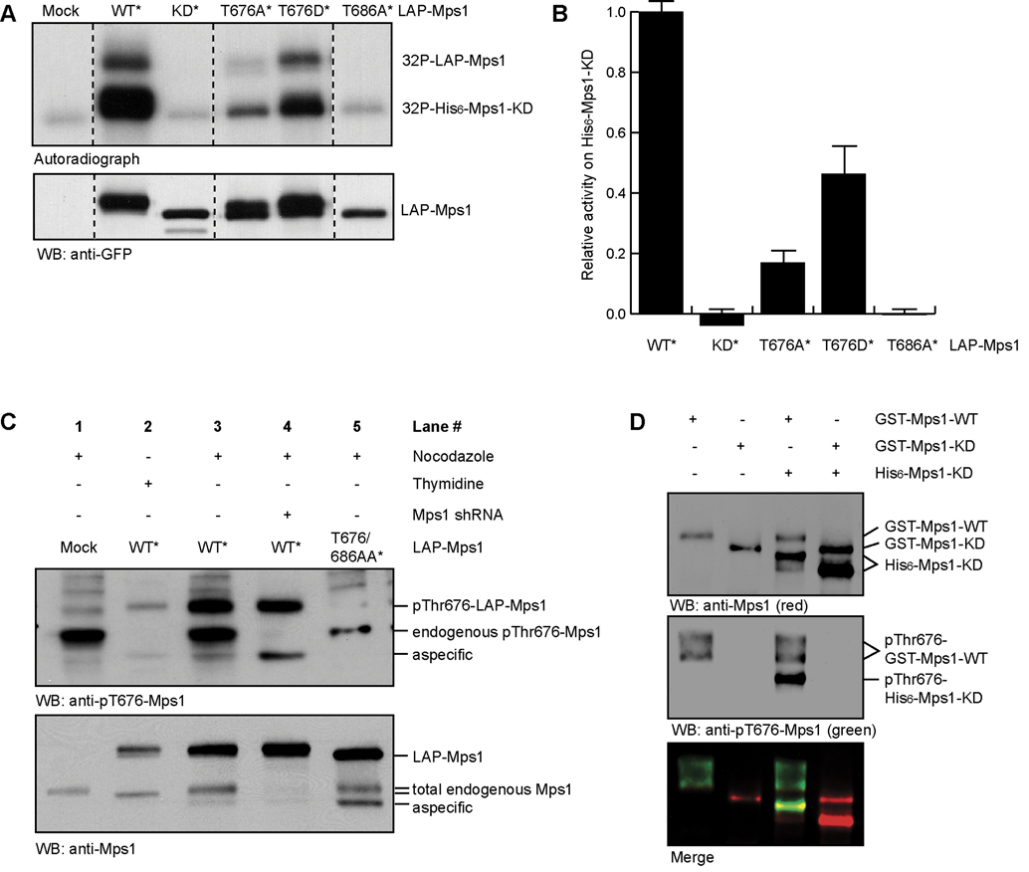
Lack of T676 auto-phosphorylation reduces Mps1 kinase activity. (A) Kinase activity of LAP-Mps1 mutants (asterisk (*) indicates mutations that confer resistance to Mps1 shRNA) extracted from mitotic U2OS cells was examined by in vitro kinase activity towards recombinant kinase-dead His-Mps1 (D664A). (B) Quantification of (A). Relative values to LAP-Mps1-WT were calculated after subtraction of the background signal derived from ‘mock’ immunoprecipitations (first lane in Fig. 3A). Average of three experiments (+/−SD). (C) Western blot showing phosphorylation specifically on T676 in lysates form U2OS cells transfected with the indicated constructs (asterisk (*) indicates mutations that confer resistance to Mps1 shRNA). (D) Western blot showing in vitro intermolecular auto-phosphorylation on T676. Combinations (as indicated) of recombinant wild-type GST-Mps1(-WT), kinase-dead GST- and His-Mps1(-KD) were subjected to a in vitro kinase reactions, after which proteins were analyzed for reactivity to pT676 antibody (green) and Mps1 antibody (red) by immunoblot.

The relationship between chromosome missegregations and Mps1 kinase activity was investigated by examining the kinase activities of the T-loop mutants compared to wild-type and kinase-dead Mps1. Since Mps1 very efficiently phosphorylates another molecule of Mps1 (Fig. S1A), kinase activities of LAP-Mps1 proteins immunoprecipitated from mitotic U2OS cells were examined by using purified recombinant kinase-dead Mps1 as substrate. The substrate (His_6_-Mps1-KD (D664A)) can subsequently be separated from the kinase (LAP-Mps1) by virtue of its faster mobility on gel. The T686A mutant showed background levels of kinase activity (Fig. 3A, 3B), confirming that this mutant is kinase-dead [19]. In agreement with the inability of T686D or T686E to restore spindle checkpoint function, these mutants were also devoid of kinase activity (data not shown). T676A however, showed reduced kinase activity, to about 20% compared to the activity of wild-type Mps1 (Fig. 3A, 3B). Phospho-mimetic mutations (Aspartic Acid, Fig. 3B; or Glutamic Acid, not shown) could restore activity to about 50% compared to wild-type. Thus although activity could not be fully restored by mimicking phosphorylation on T676, these results indicate that phosphorylation on this site plays an important role in ensuring full Mps1 activation during mitosis. To investigate whether this holds true in cells, we examined whether phosphorylation of T676 is induced specifically during mitosis by probing cell lysates with an antibody recognizing phosphorylated T676 (anti-pT676-Mps1). Lysates of cells expressing wild-type LAP-Mps1 or a LAP-Mps1-T676/686AA double point mutant, as well as lysates of cells depleted of endogenous Mps1 by RNAi were used as control for antibody specificity. Endogenous Mps1 was not phosphorylated on T676 in U2OS cells treated with thymidine to block cells in S-phase, but was highly phosphorylated on T676 in mitosis (Fig. 3C). Note that the exogenous LAP-Mps1-wild-type was partially phosphorylated on T676 and supershifted in S-phase (see lane 2, Fig. 3C), indicating that exogenous Mps1 can be activated during S-phase. Since activity of LAP-Mps1-WT immunoprecipitated from asynchronous cells is equal to that immunoprecipitated from mitotic cells (not shown), activation of exogenous Mps1 in non-mitotic cells is probably due to overexpression of the protein that can cause spontaneous auto-activation in the absence of kinetochores [18]. Probing recombinant kinase-dead Mps1 that was in vitro phosphorylated by wild-type Mps1 with the pT676-Mps1 antibody revealed that even though T676 was not identified as an autophosphorylation site by MS analysis (Fig. 1C), T676 is auto-phosphorylated in an intermolecular manner (Fig. 3D).

**Figure 4:**
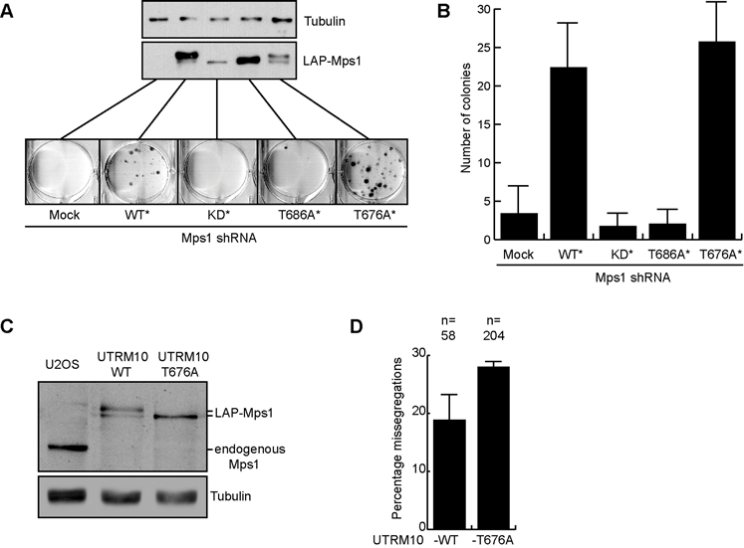
T676 phosphorylation is required for maintaining chromosomal stability but not cell viability. (A) U2OS cells were transfected as in 2B along with pBabe-puro, grown under puromycin selection for two weeks after which surviving colonies were stained. Immunoblot (anti-Mps1) shows expression levels in lysates taken 3 days after transfection of the different LAP-Mps1 mutants. (B) Quantification of the number of colonies counted in 4A, average of three experiments (+/−SD). (C) Immunoblot (anti-Mps1) showing expression levels of endogenous Mps1 and LAP-Mps1 in lysates of U2OS cells, and UTRM10 cells continuously expressing Mps1 shRNA through doxycycline treatment [14] that stably express wild-type LAP-Mps1 (UTRM10-WT) or LAP-Mps1-T676A (UTRM10-T676A). (D) Graph shows average percentages of UTRM10-T676A and UTRM10-WT cells that missegregate chromosomes as observed with live cell imaging (as in 2E), (+/−SD), n = total amount of cells.

Mps1 activity is indispensable for cell viability, but viability is maintained in CIN tumor cells that have a weakened mitotic checkpoint [5]. Since cells in which endogenous Mps1 was replaced with LAP-Mps1-T676A showed a reduction in Mps1 kinase activity and a weakened checkpoint, these cells were tested for viability in a clonogenic assay. For this, U2OS cells were transfected with Mps1 shRNA and the indicated LAP-Mps1 mutants, along with pBabe-puro. The cells were then grown in puromycin for two weeks, to allow analysis of viability of transfected cells only. As expected [14], LAP-Mps1-KD or -T686A expressing cells could not grow out to form colonies and thus proved to be unviable in the long term. Surprisingly, LAP-Mps1-T676A-expressing cells were able to grow out roughly the same amount of colonies as cells expressing wild-type LAP-Mps1 (Fig. 4A, 4B). This indicated that replacing endogenous Mps1 with an Mps1-T676A mutant did not significantly reduce cell viability. To more rigorously address the behavior of cells expressing LAP-Mps1-T676A, a cell line was created that is devoid of Mps1 but stably expresses LAP-Mps1-T676A at levels similar to the endogenous protein prior to its removal by RNAi (UTRM10-T676A) (Fig. 4C). These cells have been maintained in culture in our lab for months without obvious proliferation defects. Importantly, when imaged live, UTRM10-T676A cells displayed an increased tendency for chromosome missegregations than a similar cell line expressing wild-type LAP-Mps1 to endogenous levels (UTRM10-WT) (Fig. 4C, 4D). Thus, complete depletion from Mps1 kinase activity results in cell death but residual kinase activity of about 20 percent keeps cells viable yet induces chromosomal instability.

## Discussion

We show here that auto-activation of Mps1 by T676 auto-phosphorylation is essential for full kinase activity and maintenance of chromosomal stability, but not for cell viability. In two recent studies, T676 of human Mps1 was identified as an important phosho-site for the regulation of Mps1 activity [18], [19]. All three studies agree that Mps1 lacking this phosphorylation has strongly reduced but still clearly detectable kinase activity. Kang et al report that mitotic arrest in response to nocodazole of cells expressing T676A was reduced 2-fold [18], whereas we found no such decrease. This may be due to cell type-dependent differences in sensitivity of the checkpoint to the level of Mps1 inhibition. A more intriguing explanation, however, is the possibility that the measure that the authors of that study used to mark mitotic cells, phosphorylation of histone H3, is inadequate when analyzing effects of Mps1 inhibition on checkpoint signaling, as we have recently shown that this phosphorylation is dependent on Mps1 kinase activity in mitotic cells [14].

Kang et al found 10 phosphorylation sites on Mps1 isolated from mitotic cells [18]. Our combined data from in vivo and in vitro phosphorylated Mps1 confirms five of those sites, and these include T360, S363, S436, T676 and S821. It is unclear why there is discrepancy between the remaining five sites found in the study by Kang et al and the five additional sites from our in vivo analysis. As absence of phosphorylation of a peptide in mass spectrometric analyses is a poor indicator of lack of phosphorylation of that sequence in cells, it is quite possible that Mps1 is phosphorylated on all reported sites, totaling therefore 15 phosphorylated residues. Analysis of Mps1 using phospho-specific antibodies or other means, combined with thorough functional analysis of single point mutants is needed to pinpoint functionally relevant phosphorylations on Mps1 and distinguish actual in vivo sites from mass spec artifacts.

Cells in which endogenous Mps1 was replaced with T676A display increased frequency of chromosome missegregations as evidenced by an increase in anaphases in the presence of misaligned and/or lagging chromosomes. It is unclear what causes chromosomes to lag at anaphase in these cells. Possibly, these chromosomes are unattached but fail to activate the mitotic checkpoint due to diminished Mps1 activity. More likely, the laggers point to an inability to correct merotelic attachments. Such correction is performed by Aurora B activity, that itself is regulated by Mps1 [14]. Therefore, lagging chromosomes in T676A cells may be due to diminished Aurora B activity, resulting in persistent merotely. We have not, however, observed a detectable reduction in Aurora B activity, as T676A cells treated with MG132 properly aligned all chromosomes, suggestive of functional attachment-error-correction, and since levels of Ser7 phosphorylation of the Aurora B substrate CENP-A were indistinguishable from control (data not shown). Consistent with this, treatment of cells with low concentrations of a specific Aurora B kinase inhibitor did not cause observable inhibition of Aurora B at the level of substrate phosphorylation, chromosome alignment or cytokinesis yet resulted in persistent merotely in a significant fraction of cells [11]. Therefore, it is likely that a small, virtually undetectable, local reduction in Aurora B activity in cells expressing Mps1-T676A underlies the occasional lack of correction of a merotelic attachment in these cells.

Cells expressing Mps1-T676A arrested potently when treated with nocodazole but initiated anaphase in the presence of misaligned chromosomes. Thus, although T676 phosphorylation is not essential when the combined signals from all kinetochores is strong, it is needed for amplified checkpoint signaling from one or a few misaligned chromosomes. This supports the hypothesis that weakening of the mitotic checkpoint can cause non-lethal chromosomal instability. The contribution of such checkpoint weakening to CIN in tumor cells was recently questioned by a study in which filming of chromosome segregation in CIN tumor cells did not reveal checkpoint defects but suggested merotely as the most common way to aneuploidy in these cells [10]. Clearly in the three cell lines examined in that study, checkpoint signaling strength was sufficient to delay anaphase onset until all chromosomes had bioriented. In various other tumor cells, however, checkpoint strength was found to be diminished [5], and recent evidence from mouse models indicate that in cases where such weakening occurs, it can contribute to carcinogenesis ([23]–[25] and references in [2]). The extent of the contribution of checkpoint weakening to tumor formation is thus controversial and conclusions will await examination of chromosome segregation in large panels of cells from patient tumors. Alternatively, establishment of good biomarkers for detection of weak checkpoint signaling, such as antibodies to post-translational modifications that determine strong vs weak signaling, may facilitate this.

Despite original claims that mutations in components of the checkpoint machinery could underlie CIN [26], subsequent extensive mutational analysis of cells from many types of tumors has not confirmed this as a means to decrease the efficiency of checkpoint signaling (reviewed in [2]). Similarly, misexpression of checkpoint components has been observed [2] but it is unclear if this is the origin of CIN in those cells and there is no apparent consensus about whether up- or downregulation correlates with aneuploid status of the cells examined (e.g. [6]–[8]). For instance, disruption of the Rb tumor suppressor pathway causes CIN by upregulating Mad2 whereas inactivation of the REST tumor suppressor causes CIN by decreasing expression of Mad2 [27], [28]. Similarly, mice either overexpressing Mad2 or underexpressing Mad2 develop cancer, albeit that affected organs, tumor types and latency periods differ [29], [30]. It is thus possible that checkpoint weakening in tumor samples at the molecular level is caused by altered activity of checkpoint components or by altered post-translational modifications that contribute to efficiency of the mitotic arrest.

The data presented here place Mps1 as a central activity to prevent chromosomal instability. Reduced Mps1 kinase activity allows weakened mitotic checkpoint signaling as well as lagging chromosomes at anaphase, two major defects suggested to contribute to CIN in tumor cells [5], [10]. Phosphorylation of T676 in Mps1 provides the first specific post-translational modification in human cells as a cause for this. Many more such modifications may exist and it will be interesting to examine if any are affected in CIN tumor cells.

## Materials and Methods

### Cells

U2OS cells were grown in DMEM with 8% FBS, supplemented with pen/strep. UTRM10 cells [14] were grown similarly with the exception that tet-approved FBS was used. UTRM10-676A and -WT cells were created by transfecting UTRM10 cells (Effectene, Qiagen) with LAP-Mps1-T676A or -WT and selecting for survivors by continuous culture in 1 µg·ml^−1^ doxycycline (Sigma). Clones were subsequently chosen based on expression of the LAP-tagged Mps1 protein and absence of endogenous Mps1. Thymidine (2.5 mM), nocodazole (200 ng·ml^−1^) and puromycin (1 µg·ml^−1^) were all from Sigma.

### Plasmids, cloning and transfections

pSuper-mock, pSuper-Mps1, pcDNA3-LAP-Mps1-WT and -D664A have been described [14]. Point mutations were generated by site-directed mutagenesis. All sequences were verified by automated sequencing. Mps1 was depleted from U2OS cells using shRNA and replaced by RNAi-resistant LAP-Mps1-wild-type, -kinase-dead (D664A), -T676A, or -T686A, by transfection with calcium phosphate (for details on this protein replacement assay see [14]).

### Antibodies

pT676-Mps1 antibodies were raised in rabbits using the peptide CMQPDTpTSVVKDS coupled to KLH as antigen (Eurogentec). Phospho-specific polyclonal antibody used in this study was affinity purified using the described peptide after removal of non-phospho-specific antibodies with an unphosphorylated version of the peptide. Anti-Mps1-NT was from Upstate Biotechnology, ACA was from Cotrex Biochem and α−Tubulin was from Sigma. Anti-GFP was a custom polyclonal antibody.

### Identification of mitotic phosphorylation sites

Cells were blocked in mitosis by treatment with nocodazole for 18 hrs and harvested by mitotic shake-off. LAP-Mps1 was isolated by tandem affinity purification [17] and phosphorylated residues were identified using mass spectrometry as described [31].

### In vitro recombinant kinase assay

Wild-type GST-Mps1 was expressed and purified from High-Five insect cells using the Life Technologies Bac-to-Bac system. Recombinant active Mps1 was incubated in kinase buffer (50 mM Tris pH 7.4, 10 mM MgCl_2_, 0.5 mM DTT, 40 µM ATP) at 30°C for 30 minutes. (Auto-)phosphorylated residues were identified using mass-spectrometry as described [14].

### IP kinase assay

U2OS cells transfected with the various LAP-Mps1 alleles were released from a 24 hours thymidine block into nocodazole for 18 hours, harvested and lysed in 150 mM NaCl, 50 mM Hepes pH7.5, 5 mM EDTA and 0.1% NP40. LAP-Mps1 was removed from the lysates with S-protein agarose (Novagen) and tested for kinase activity towards recombinant kinase-dead His-Mps1 (D664A) in kinase buffer (50 mM Tris pH 7.5, 10 mM MgCl_2_, 0.5 mM DTT, 40 µM ATP) using [γ-32P]-ATP.

### Flow cytometry and colony outgrowth

U2OS cells were released from a 24 hours thymidine block into nocodazole for 18 hours, harvested and fixed in 70% ice-cold ethanol for 24 hours. Fixed cells were immunostained with anti-Mpm2 (Upstate Biotechnology) to determine the fraction of mitotic cells. Flow cytometric analysis of transfected cells was done based on Spectrin-GFP co-transfection. Colony outgrowth assays were done essentially as described [32].

### Immunofluorescence

Immunostaining experiments were done as described [14]. For alignment analysis, U2OS cells plated on 15 mm coverslips were treated and fixed with 4% Shandar Zinc Formal-Fixx™ (Thermo electron corporation) for 20 minutes. Fixed cells were stained with DAPI and the percentage of cells with all chromosomes aligned to the metaphase plate was determined using fluorescence microscopy [14].

### Time-lapse video microscopy

Mps1 was depleted from U2OS cells using shRNA and replaced with RNAi-insensitive LAP-Mps1-WT or -T676A, and mitotic progression was followed with live cell imaging as described [14]. DNA was visualized by co-transfecting H2B-EYFP.

## Supporting Information

Figure S1(A) Wild-type GST-Mps1 (WT) cross-phosphorylates kinase-dead His-Mps1 (KD) in vitro as measured by 32P incorporation from [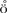-32P]-ATP. (B) U2OS cells were transfected with the indicated constructs (asterisk (*) indicates mutations that confer resistance to Mps1 shRNA). Cells were treated as in (2B) and the percentage of mitotic cells was determined as in (2B).(0.69 MB TIF)

Movie S1 Mps1 was depleted from U2OS cells using shRNA and replaced with RNAi-insensitive LAP-Mps1-T676A, and mitotic progression was followed with live cell imaging as described [14]. DNA was visualized by co-transfecting H2B-EYFP.
(0.07 MB MOV)

Movie S2 Mps1 was depleted from U2OS cells using shRNA and replaced with RNAi-insensitive LAP-Mps1-T676A, and mitotic progression was followed with live cell imaging as described [14]. DNA was visualized by co-transfecting H2B-EYFP.
(0.12 MB MOV)
