# Supplementary figures and images for: Chromosomal Instability by Inefficient Mps1 Auto-Activation Due to a Weakened Mitotic Checkpoint and Lagging Chromosomes
Source: PLoS One. 2008 Jun 11;3(6):e2415. doi: 10.1371/journal.pone.0002415 (PMC2408436; doi:10.1371/journal.pone.0002415)

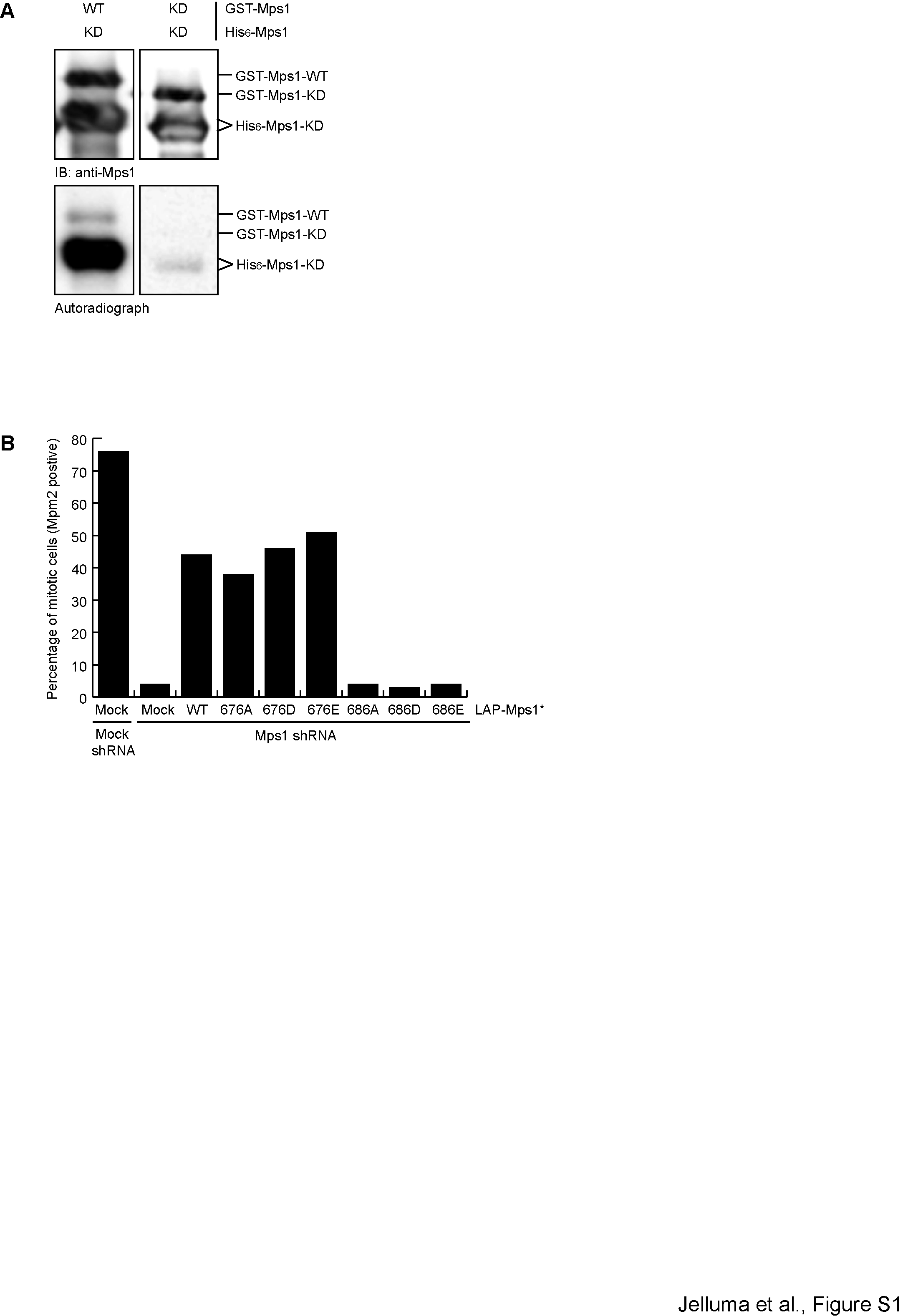

Supplement: Figure S1 — (A) Wild-type GST-Mps1 (WT) cross-phosphorylates kinase-dead His-Mps1 (KD) in vitro as measured by 32P incorporation from [-32P]-ATP. (B) U2OS cells were transfected with the indicated constructs (asterisk (*) indicates mutations that confer resistance to Mps1 shRNA). Cells were treated as in (2B) and the percentage of mitotic cells was determined as in (2B). (0.69 MB TIF) [file pone.0002415.s001.tif]
